# Beta-Blocker Therapy and Hemophagocytic Lymphohistiocytosis: A Case Report

**DOI:** 10.4061/2010/912757

**Published:** 2010-06-20

**Authors:** C. Müller, L. B. Mänhardt, C. Willaschek, E. M. Schneider, E. A. Stuth, R. Buchhorn

**Affiliations:** ^1^Department of Pediatrics, Caritas Krankenhaus, 97980 Bad Mergentheim, Germany; ^2^Section of Experimental Anaesthesiology, Department of Clinical Anaesthesiology, University Hospital, 89075 Ulm, Germany; ^3^Department of Anaesthesiology, Medical College of Wisconsin, Milwaukee, WI 53226-4801, USA; ^4^Klinik für Kinder- und Jugendmedizin, Caritas Krankenhaus, Uhlandstraße 7, 97980 Bad Mergentheim, Germany

## Abstract

*Objective*. The aim of this paper is to describe a fatal case of hemophagocytic lymphohistiocytosis (HLH) in a patient with severe heart failure, who was treated with low-dose propranolol. *Patient and Interventions*. We report on a 7-month-old boy with Downs syndrome who was born with an unbalanced, left dominant atrioventricular septal defect and aortic coarctation. Despite coarctation repair and pulmonary artery banding he developed intractable heart failure and fever of unknown origin. Since he remained in heart failure he received a trial of low-dose propranolol to stabilize his cardiopulmonary status, which resulted in unexpected immunomodulatory effects. *Measurements and Main Result*. Immunoactivation was evidenced by high concentrations of procalcitonin, soluble CD 25, tumor necrosis factor *α*, and interleukin 6 and 8. Propranolol resulting in hepatic compromise as indicated by high lactate dehydrogenase and alanine aminotransferase levels. A therapeutic switch from propranolol to the *β*_1_-receptor blocker metoprolol appeared to be instrumental in hemodynamic improvement and allowed discharge from hospital. However, the infant ultimately died from secondary inflammatory reactivation and intractable pulmonary obstructive disease. The autopsy results revealed HLH. *Conclusion*. Our case describes HLH secondary to heart failure and Downs syndrome. In this highly activated inflammatory state the beneficial hemodynamic effects of propranolol may be accompanied by immunomodulatory effects and the risk of acute liver failure. HLH occurs with a distinct pathophysiology, and specific treatment might be mandatory to increase the chance of survival.

## 1. Introduction

HLH is a life-threatening condition which may be difficult to distinguish from severe sepsis [1]. We report an infant who presented with a complex congenital heart defect, Downs syndrome, and fever of unknown origin who appeared to be in intractable heart failure but the autopsy results revealed HLH. While in apparent intractable heart failure by clinical signs, the infant received a trial of low-dose propranolol, which resulted in immunomodulatory effects characterized by septic fever, elevated laboratory parameters (Table 1), and multiorgan dysfunction. The effects of this non selective *β*-blocker on a highly activated inflammatory state provide us with an opportunity to gain pathophysiological insights into interactions between the adrenergic system and the inflammatory response. 

## 2. Case Report

We report on a 7-month-old boy with Downs syndrome who was born with an unbalanced left dominant atrioventricular septal defect and aortic coarctation. Initially he underwent aortic coarctation repair. However, despite good restitution of the systemic obstruction he could not be weaned from mechanical ventilation and subsequently underwent pulmonary arterial banding, which reduced pulmonary arterial pressure to 50% of systemic pressure and the pulmonary-to-systemic flow ratio to 1.5 : 1. Also a plication operation was performed for left-sided diaphragmatic paralysis. Supportive pharmacotherapy consisted of digoxin, captopril, spironolactone, and high-dose furosemide. In addition, a gastro-duodenal tube (G-Tube) was placed for feeding because of vomiting and poor oral intake. The hospital course was complicated by more than 10 endotracheal intubations for recurrent respiratory failure, which appeared to be triggered by exacerbations of an inflammatory disease process of unknown origin. During this time the infant underwent multiple courses of antibiotic treatment. Despite multiple diagnostic efforts, the cause of the recurrent sepsis-like exacerbations could not be determined. 

The hemodynamic status was characterized by good cardiac performance of the functionally single ventricle despite persistent pulmonary hypertension (Table 1). There was clear evidence for ongoing pulmonary vascular disease with pulmonary hypertension and need for oxygen supplementation. In this situation, stage 2 palliation (superior cavopulmonary connection, bidirectional Glenn shunt) of his single ventricle defect was considered contraindicated. Since we hypothesized that the recurrent clinical symptoms of heart failure were caused by neurohormonal and inflammatory dysfunction we started a trial of low-dose beta-blocker therapy with 0.3 mg/kg/day propranolol (0.1 mg/kg per dose) by G-Tube in accordance with the German guidelines for congenital heart defects in children [2, 3]. 

During the next fever episode the infant developed signs of hepatic failure and pulmonary edema and required intubation with mechanical ventilation as well as intravenous antibiotics and aggressive diuretic treatment. Since the hemodynamic and cardiac function improved, propranolol therapy was not stopped. He recovered from this initial episode of systemic inflammatory response syndrome (SIRS) (see SIRS I in Table 1) within 3 days. He was extubated without dyspnea and began to tolerate oral feeding. However the fever continued unabated and when we stopped antibiotic coverage to collect blood cultures he decompensated with signs of an exacerbation of the SIRS II (see Table 1). 

Furthermore we switched the medication to the selective *β*_1_-blocker metoprolol. The patient stabilized quickly again but the fever continued. Since all blood cultures remained negative, we hypothesized that the source of recurrent endotoxemia may be gastrointestinal. One week later we decided to remove as many potential sources of infection as possible. We removed not only the central venous catheter but also the G-Tube. Subsequent blood cultures and the central venous catheter cultures were negative, but resistant E. coli was recovered from the jejunal end of the feeding tube. The infant improved with oral treatment with cotrimoxazole. His supplemental oxygen requirements ceased during his oxygen saturation rose to 90% and the pulmonary arterial pressure decreased to the normal range. After six months of intensive care, including 12 intubations for respiratory failure, he was discharged home. Two days later he was readmitted for fever and decreasing oxygen saturations due to bronchospasm. An etiology of the bronchospasm could not be ascertained. Despite intensive therapy with systemic and inhalational *β*_2_-adrenergic agonists, theophylline, and high-dose prednisolone, the bronchospasm persisted and the infant's condition deteriorated. Metoprolol was discontinued and mechanical ventilation was reinitiated. A subsequent event of pulmonary hypertensive crisis ensued and unfortunately the patient expired (SIRS III). Post mortem the results of a bone marrow aspirate showed hemophagocytosis and the diagnosis of HLH was verified by a highly elevated ferritin and soluble CD25 receptor levels. Especially the cytokine profiles confirmed the diagnosis of HLH. Epstein-Barr virus infection could be excluded by polymerase chain reaction.

## 3. Discussion

HLH can mimic heart failure with signs of hepatosplenomegaly, dystrophy, pulmonary, and systemic congestion resistant to diuretic therapy. HLH in our patient was not recognized earlier, because this entity has not been described in the context of heart failure [4]. Immunoactivation due to heart failure has been reported in adults with left ventricular dysfunction [5] and in children with congenital heart disease [6]. We have previously reported elevated cytokine levels in infants with heart failure and the beneficial effect of propranolol [7]. As shown in Table 1, immunoactivation in our patient was characterized by high procalcitonin and cytokine levels. This constellation together with normal high sensitivity C-reactive protein values and highly elevated probrain natriuretic peptide (NT-Pro-BNP) was indicator of an SIRS [8] of a non-infectious etiology. In this situation low-dose propranolol led to an unexpected hepatic compromise as indicated by high lactate dehydrogenase and alanine aminotransferase levels unrelated to the deterioration of global hemodynamics. Severe liver cell necrosis was also described during *β*_2_-blockade in systemic inflammatory response syndrome in animals [9]. 

The observed hyperinflammatory response with liver cell necrosis appears to be *β*_2_-receptor mediated since we did not observe similar elevations of lactate dehydrogenase and alanine aminotransferase levels during therapy with the selective *β*_1_-antagonist metoprolol. However, in whole blood samples from patients with heart failure bisoprolol, another *β*_1_-selective betablocker, but not metoprolol or carvedilol augments LPS-stimulated TNF-*α* production [10]. These results are rather curious and need further investigation. It is also possible that the nonspecific *β*-blocker impaired NK activation and thus promoted HLH [11]. 

It is becoming evident that sympathetic nervous system activation may play a dual role during any inflammatory response. While it clearly has profound anti-inflammatory effects during systemic inflammation, a recently published study suggests that the local inflammatory response can be immensely amplified by local cell-derived catecholamine production. Thus, catecholamines enhance cytokine release by macrophages via activation and translocation of NF*κ*B, demonstrating that catecholamines are powerful cellular hormones that self-regulate the activation level and the inflammatory potential of inflammatory cells [12]. Since HLH is causally related to NK dysfunction and concurrent viral infections in the majority of cases, the activation of NK function demonstrated by *β*-receptor blockade in a surgical and cancer model is noteworthy [13]. 

## 4. Conclusion

Our case describes an infant with secondary HLH due to heart failure and Downs syndrome. In this highly activated inflammatory state the beneficial hemodynamic effects of propranolol may be accompanied by unexpected immunomodulatory effects and the risk of acute liver failure. Before institution of *β*-blocker therapy in patients with heart failure associated with inflammation, HLH has to be excluded by clinical and laboratory findings focussing on soluble CD25 [14] and ferritin measurement [15]. HLH occurs with a distinct pathophysiology, and specific treatment is mandatory to increase the chance of survival [16]. 

## Figures and Tables

**Table 1:** 

Parameter	Normal Value	Baseline	SIRS I	SIRS II	Discharge	SIRS III
Timeframe (days)			3	4	2	18
Age (weeks)			23	24	27	29

Cardiovascular changes						
Heart rate (1/min)		130	110	100	100	150
Fractional shortening (%)		45	45	32	59	36
LV Pressure (mmHg)^§^		101	79	79	71	63
Mean arterial pressure (mmHg)		86	65	74	60	50
Pulmonary artery pressure_sys_ (mmHg)^#^		63	61	48	30	40

Hepatic changes						
GOT_max _ (U/l)	0–35	319	28839	3778	107	323
LDH_max _ (E/l)	0–250		220000	9097	530	2753
Bilirubin_max _ (mmol/l)	0.4–1.2		2.8	9.51	6.16	7.06

Renal changes						
Creatinine_max _ (mg/dl)	0.70–1.20	0.45	0.62	1.94	0.67	1.57
Urea_max _ (mg/dl)	11–55		75	133	122	210

Coagulation						
Thrombocyte_min _ (1000/l)	140000–400000	690000	153000	88000	61000	58000
INR_max _ (Ratio)	0.8–1.25		2.11	3.24	1.34	1.47

Inflammatory markers						
White blood cell_max _ (1000/l)	3.6–10.0	16.7	28.280	16.999	12.800	3.690
Normoblast absolute (1000/l)	None	0	22.220	2.101	0	16.810
Procalcitonin_max _ (pg/ml)	0.1–0.5	5.61	13.67	85.78	2.9	420
sCD25 (U/ml)*	<1000	2220	2160			3916
TNF-*α* (pg/ml)*	<3	90.4	104.8			98.4
IL-8 (pg/ml)*	<3	152	346			328
IL-6 (pg/ml)*	<6	72	108			55.6
IL-1*β* (pg/ml)*	<5	2.9	4.0			1.68
hs CRP_max _ (mg/dl)	<1.0	0.00	0.21	0.83	0.21	0.00
NT-Pro-BNP (pg/ml)	0–85		6374	31127	31689	71485
Ferritin (*μ*g/l)*	20–400	33016	27516	94099	419	3676
Triglyceride (mg/dl)	35–180			1349	222	327
Serum Calcium_min _ (mmol/l)	2.15–2.75	1.81	2.23	1.61	2.54	2.05

*: measurements from stored plasma

^§^: left ventricular pressure estimated from Doppler measurements of mitral valve regurgitant jet velocity

^#^: systolic pulmonary artery pressure estimated using Doppler measurements across pulmonary artery band:

Pulmonary artery pressure_sys_ = LV pressure – PAB gradient.

## Appendix

A trial of beta-blocker therapy was initiated after receiving full-informed consent from the parents. The ethic committee waived the need for approval.
